# On-field optical imaging data for the pre-identification and estimation of leaf deformities

**DOI:** 10.1038/s41597-022-01795-4

**Published:** 2022-11-12

**Authors:** Sm Abu Saleah, Ruchire Eranga Wijesinghe, Seung-Yeol Lee, Naresh Kumar Ravichandran, Daewoon Seong, Hee-Young Jung, Mansik Jeon, Jeehyun Kim

**Affiliations:** 1 School of Electronic and Electrical Engineering, College of IT Engineering, Kyungpook National University, 80, Daehak-ro, Buk-gu, Daegu, 41566 South Korea; 2 Department of Materials and Mechanical Technology, Faculty of Technology, University of Sri Jayewardenepura, Pitipana, Homagama, 10200 Sri Lanka; 3 School of Applied Biosciences, Kyungpook National University, 80, Daehak-ro, Buk-gu, Daegu, 41566 South Korea; 4 Center for Scientific Instrumentation, Korea Basic Science Institute, 169-148, Gwahak-ro Yuseong-gu, Daejeon, 34133 South Korea

**Keywords:** Electrical and electronic engineering, Imaging and sensing, Biophotonics

## Abstract

Visually nonidentifiable pathological symptoms at an early stage are a major limitation in agricultural plantations. Thickness reduction in palisade parenchyma (PP) and spongy parenchyma (SP) layers is one of the most common symptoms that occur at the early stage of leaf diseases, particularly in apple and persimmon. To visualize variations in PP and SP thickness, we used optical coherence tomography (OCT)-based imaging and analyzed the acquired datasets to determine the threshold parameters for pre-identifying and estimating persimmon and apple leaf abnormalities using an intensity-based depth profiling algorithm. The algorithm identified morphological differences between healthy, apparently-healthy, and infected leaves by applying a threshold in depth profiling to classify them. The qualitative and quantitative results revealed changes and abnormalities in leaf morphology in addition to disease incubation in both apple and persimmon leaves. These can be used to examine how initial symptoms are influenced by disease growth. Thus, these datasets confirm the significance of OCT in identifying disease symptoms nondestructively and providing a benchmark dataset to the agriculture community for future reference.

## Abstract (summary)

**Table:** 

Measurement(s)	Apple and persimmon leaf layer thickness
Technology Type(s)	Optical coherence tomography imaging and MATLAB programs

## Background & Summary

Plant and fruit diseases are impairments of the normal state of a plant that interrupt or modify its vital functions. Apple is one of the most widely produced fruits globally, whereas persimmon is mainly cultivated in East Asian countries, such as Korea, Japan, and China^1,2^. Apple scab, marssonina leaf blotch, black rot canker, and alternaria leaf spot/blight are examples of diseases of apple, in which symptoms can be identified on the leaves after disease progression^3–5^, which reduce the quantity and quality of the produce. Circular leaf spot is the most damaging pathogenic disease in persimmon cultivation^6^, causing discoloration and defoliation of diseased leaves and resulting in massive economic losses^7,8^. In most cases, the disease can be treated and controlled if the symptoms are identified at an early stage^9,10^.

**Fig. 1:**
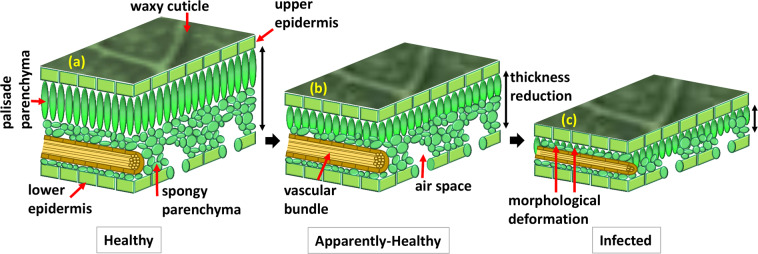
A schematical illustration of leaf inner morphology and its gradual changes from healthy to infected state.

The initial symptoms of these diseases occur mainly on the leaf subsurface, which is a complex organ comprised mostly of palisade parenchyma (PP) and spongy parenchyma (SP), crossed by vascular tissue, and surrounded by two epidermises^11^. The PP has regular-shaped cells near the upper surface of the leaf, whereas the SP is less well-organized and located near the lower epidermis of the leaf^12,13^. These layers are crucial in manufacturing food, gas exchange, and water evaporation. Therefore, the pre-identification of plant diseases by detecting abnormalities of PP and SP is important for appropriate timing control by reducing damage and production cost and increasing production. The schematic shown in Fig. 1 elaborates the inner morphology of leaf specimens illustrating gradual structural changes and thickness reduction with disease progression between PP and SP.

Several methods have been introduced for the early detection of leaf diseases. Visual inspection is commonly used; however, it is subjective, inefficient, time-consuming, labor-intensive, and costly in the early stages of infection^14–16^. In contrast, physiological, biological, serological, and molecular tests are laboratory-based methods used for the identification of plant disease^17–20^. Polymerase chain reaction, enzyme-linked immunosorbent assay, and histological sectioning are some laboratory test-based plant disease evaluation methods that are destructive, complex, time-consuming, and expensive^21,22^. To compensate for these drawbacks in plant disease detection, noninvasive techniques, such as image processing^23–26^, terrestrial laser scanning^16^, sonic tomography^18^, electronic nose^27^, microfocus X-ray fluorescence^28^, GanoSken technology^29^, and spectroscopy^30^, have gained much attention. However, a long setup process, complexity, high cost, sensitivity to environmental change, low selectivity, and highly specific software requirements^20,31^ are some drawbacks of these techniques. Noninvasive morphological and structural imaging of plant materials has been performed using ultrasound^32^, X-ray^33^, magnetic resonance imaging^34^, and positron emission tomography imaging^35^. However, these imaging techniques have low image resolution and long image acquisition time^36–39^. Therefore, a noninvasive optical imaging technique is required for the early detection of plant disease progression by investigating subsurface structures of leaf specimens.

High-resolution optical coherence tomography (OCT) is a noninvasive imaging technique that provides cross-sectional images using a nonionizing broadband light source^40^. The image resolution of OCT is 1–15 μm (10–100 times better than ultrasound)^41^. OCT has been widely used in various fields, including medical diagnosis^42,43^, dentistry^44,45^, dermatology^46,47^, tissue imaging^48,49^, agriculture^50,51^, and industrial applications^52,53^. Because OCT imaging depth (1.5–2 mm) is suitable for the micrometer-scale visualization of the internal structure of plant leaves, OCT-based agricultural disease detection studies have established a solid platform to confirm the applicability of OCT in plant disease inspection^51,54–62^.

To elaborate the potential merits of OCT for inspecting plant diseases, various coordinates of OCT images and an optical signal intensity-based depth profile algorithm are presented in this study. The developed algorithm was incorporated to set a threshold for the pre-identification of PP and SP layer abnormalities in persimmon and apple leaf specimens by assessing OCT cross-sectional images. The developed depth profile algorithm was applied to cross-sectional OCT images to quantitatively evaluate the inner layer structure of leaf specimens. The obtained data sets revealed a gradual thickness reduction between PP and SP layers in healthy, apparently-healthy, and infected specimens of persimmon and apple leaves. A threshold value was set based on the thickness differences obtained from the collected datasets for detecting early abnormalities in persimmon and apple leaves, which can be used to assess plant leaf diseases in the future.

## Methods

### Optical imaging modality

**Fig. 2:**
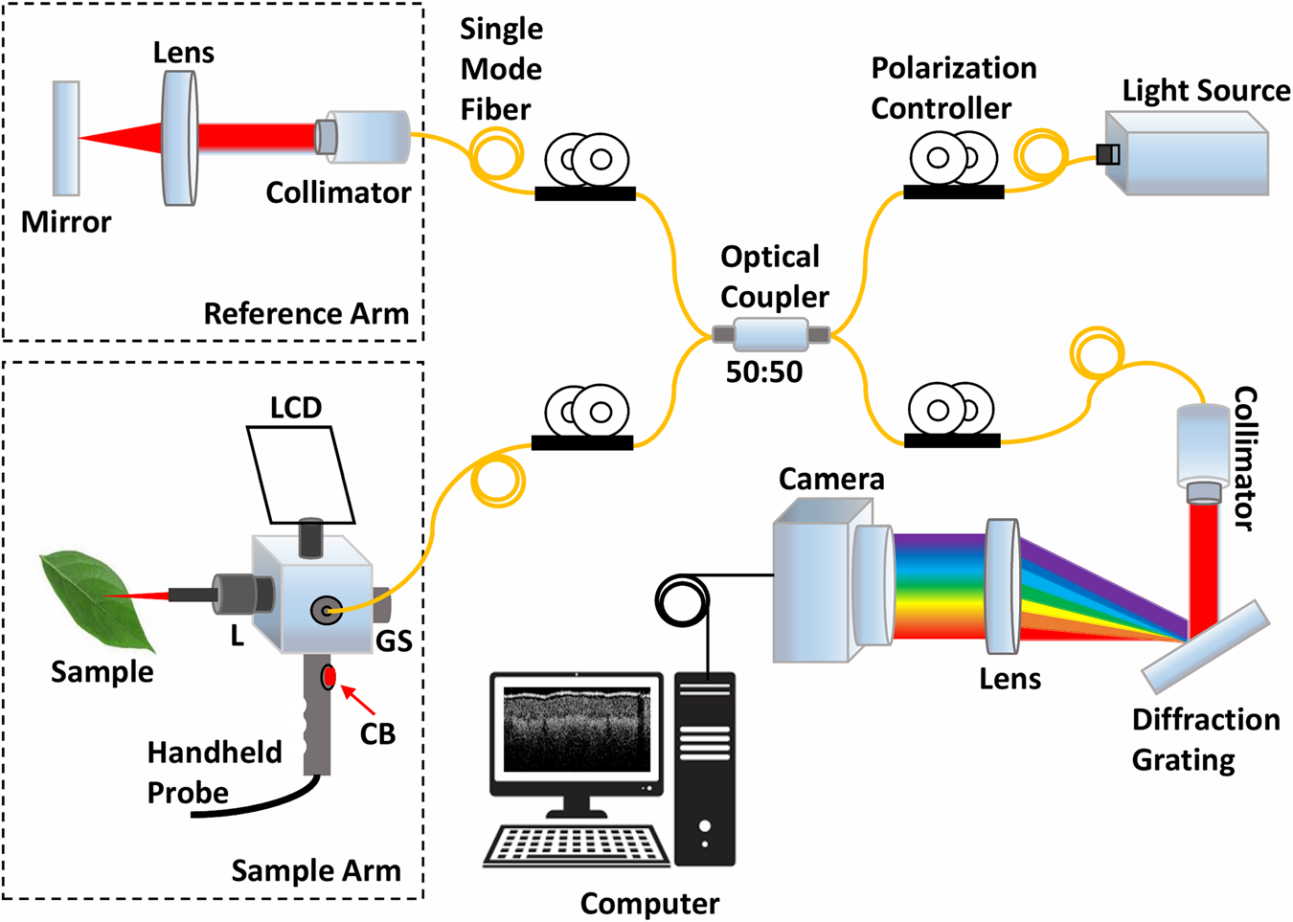
Schematic of the spectral-domain optical coherence tomography (SD-OCT) system used for data acquisition. CB: capture button; GS: galvo-scanner; L: lens; LCD: liquid crystal display.

The schematic of the optical configuration of SD-OCT used in this study is shown in Fig. 2. The system was equipped with a broadband light source (EXS210068-01, Exalos, Switzerland) with a central wavelength of 850 nm, full width at half maximum bandwidth of 55 nm, and average output power of 5 mW. A galvanometer-based optical scanner (GVS002, Thorlabs, USA) and a 1-inch object lens (AC254-030-B, Thorlabs, USA) were used in the handheld probe-based sample arm for transversely scanning the sample. The reference arm was identically composed of a collimator (F260APC-B, Thorlabs, USA), lens (AC254-030-B, Thorlabs, USA), and mirror (PF10-03-P01, Thorlabs, USA). The ratio of the fiber couplers (TW850R5A2, Thorlabs, USA) used in this system was 50:50. The back-scattered signals from the sample and reference arms were coupled together through the coupler and transferred to a customized spectrometer. The spectrometer was calibrated using a previously described method^40^. A 2048-pixel line scan camera (spL2048-140 km, Basler, Germany) was used for image acquisition. A miniature LCD panel was connected to the handheld scanner for real-time OCT image preview, and a 1.5 m handheld probe with a capturing button was connected to the handheld scanner to save the OCT image. The axial and lateral resolutions of this OCT system were 5.1 and 11 μm, respectively, when measured in air.

### Plant leaf specimens

**Fig. 3:**
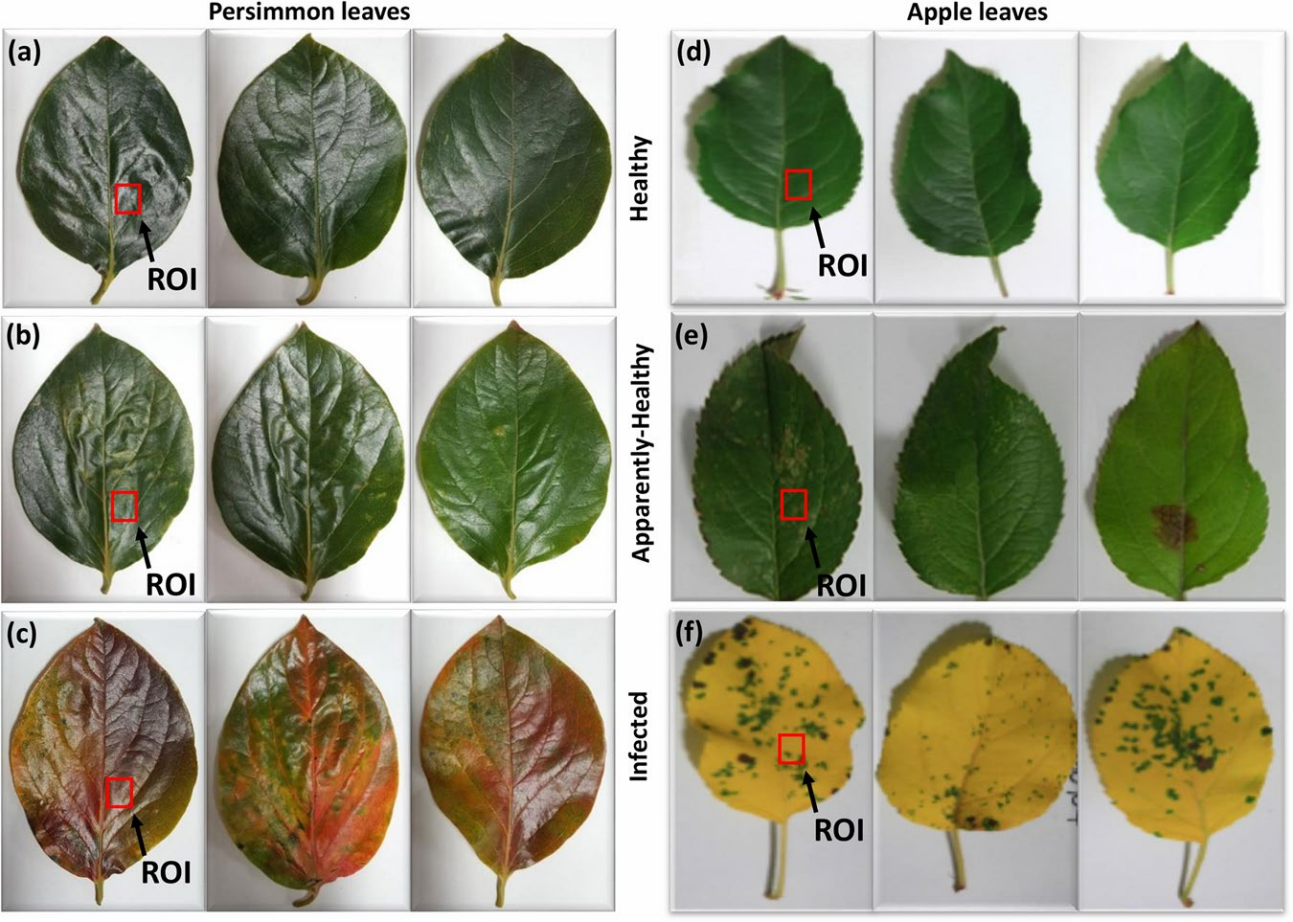
Photographs of healthy, apparently-healthy, and infected persimmon and apple leaves. (**a**–**c**) show healthy, apparently-healthy, and infected persimmon leaves, respectively; (**d**–**f**) show healthy, apparently-healthy, and infected apple leaves, respectively. UE: upper epidermis, PP: palisade parenchyma, SP: spongy parenchyma.

The photographs of persimmon and apple leaf specimens are presented in Fig. 3. Healthy, apparently-healthy, and infected leaves of the persimmon tree are shown in Fig. 3(a–c), respectively. Figure 3(d–f) represent healthy, apparently-healthy, and infected leaves of the apple tree, respectively. The healthy and apparently-healthy leaves of both persimmon and apple trees have a similar appearance; however, the infected leaves of both trees were discolored. The visual inspection method was unable to provide early detection of leaf abnormalities, whereas the analysis of the internal structure of healthy, apparently-healthy, and infected leaves of persimmon and apple trees could be performed by assessing OCT cross-sectional images.

The disease name and the causative agents of the persimmon and apple leave employed in this study are given in Table 1:

**Table 1:** The disease name and the causative agents of the persimmon and apple leaves.

	Persimmon	Apple
Disease name	Circular leaf spot	Apple blotch
Causal agent	Mycosphaerella nawae	Diplocarpon coronariae

### Leaf inspection algorithm for persimmon and apple

**Fig. 4:**
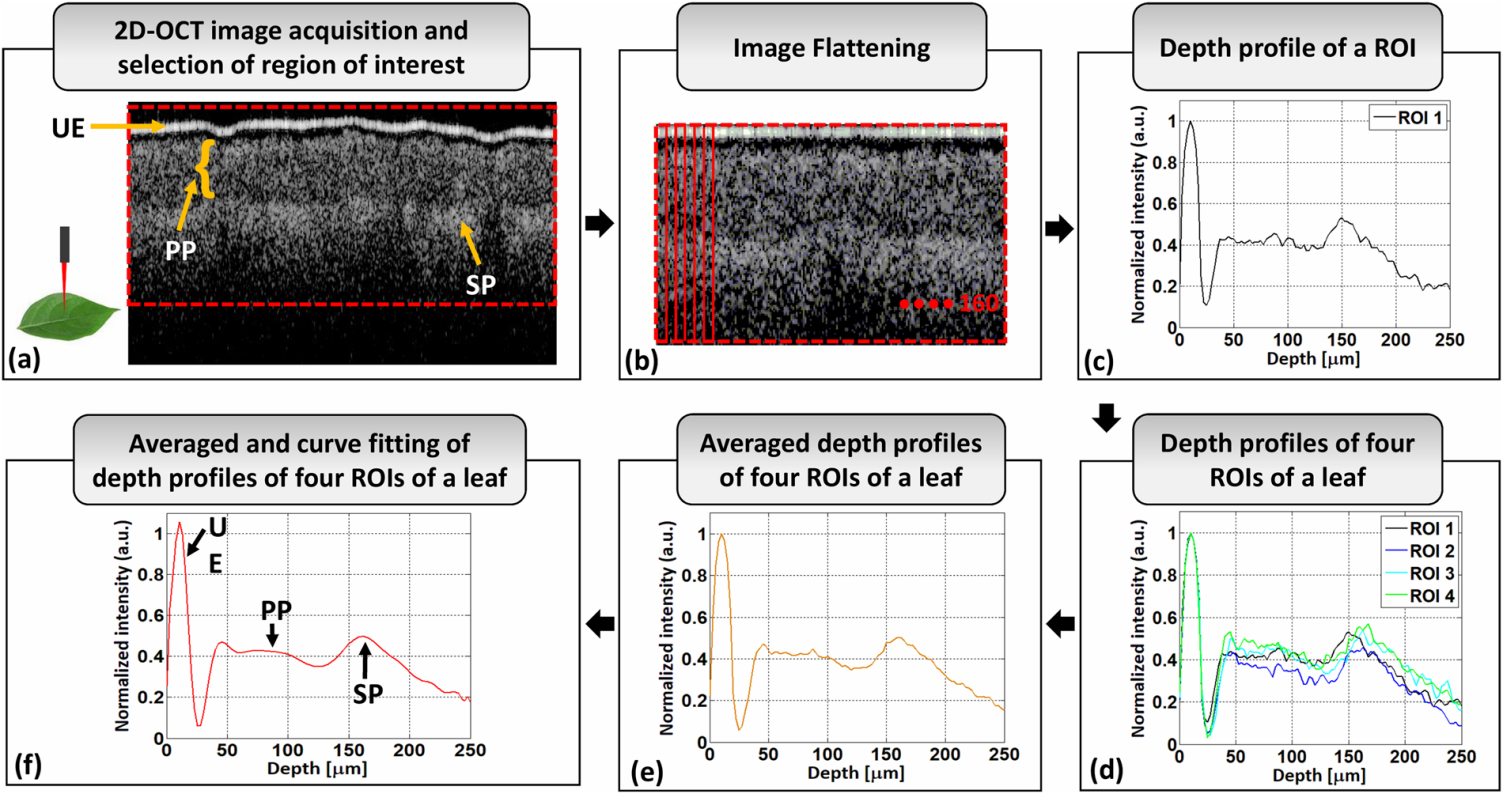
Algorithm for measuring persimmon leaf layer thickness. (**a**) Leaf-scanning position with an optical scanner and 2D cross-sectional image of a persimmon leaf with different layers. (**b**) 2D cross-sectional image after flattening. (**c**) The depth profile of a region of interest (ROI) of a single leaf. (**d**) Depth profiles of four ROIs of a single leaf. (**e**) Average depth profile of four ROIs of a single leaf. (**f**) Curve-fitted depth profile of four ROIs of a single leaf. UE: upper epidermis, PP: palisade parenchyma, SP: spongy parenchyma.

The algorithm for measuring the thickness between the PP and SP layers of persimmon using depth intensity profile is depicted in Fig. 4. The optical scanner, with the scanning position of the leaf, and the corresponding 2D OCT image of persimmon leaf with the upper epidermis (UE), PP, and SP layers are depicted in Fig. 4(a). A software program coded in Matlab (Mathworks, USA) was used to search for the intensity peak in the depth direction for the depth intensity profile analysis. After the 2D cross-sectional OCT image was loaded, RGB to grayscale conversion was performed. Then, a window was selected from the unflattened 2D OCT image as a region of interest (ROI), marked with a red dotted box in Fig. 4(a), to apply a peak search algorithm during the depth intensity profile analysis. The peak search algorithm consecutively detected the highest intensity in each A-scan line. The unflattened 2D cross-sectional image contains the highest intensity index points at different positions in the lateral direction due to the physical structure of a leaf sample. Therefore, to get a flattened image, index positions with high intensity should be adjusted and matched linearly. Figure 4(b) shows the flattened image of the 2D OCT images shown in Fig. 4(a), with the thickness measuring ROI marked by the red dotted rectangle. A total of 160 A-scan lines were taken from the selected ROI, shown in the flattened image, and then summed up and averaged to get a single depth intensity profile for measuring the thickness of the persimmon leaf. The A-scan intensities were normalized by dividing them by their maximum values to obtain a stable intensity profile. Moreover, a median filter was used in the software program to compensate for the speckle noise to obtain a noise-free and smooth intensity plot. Figure 4(c) shows a single depth intensity profile of a selected ROI of a persimmon leaf. To obtain a more reliable depth intensity profile of a leaf, four ROIs were selected randomly from different positions on that leaf. Figure 4(d) shows the depth intensity profiles of four ROIs of a persimmon leaf. The depth intensity profiles of four ROIs of a leaf were then summed and averaged to get a reliable depth intensity profile of a leaf in a single profile, as shown in Fig. 4(e). The average depth intensity profile of four ROIs was then curve fitted to obtain a smooth depth intensity profile of a single persimmon leaf, as shown in Fig. 4(f).

**Fig. 5:**
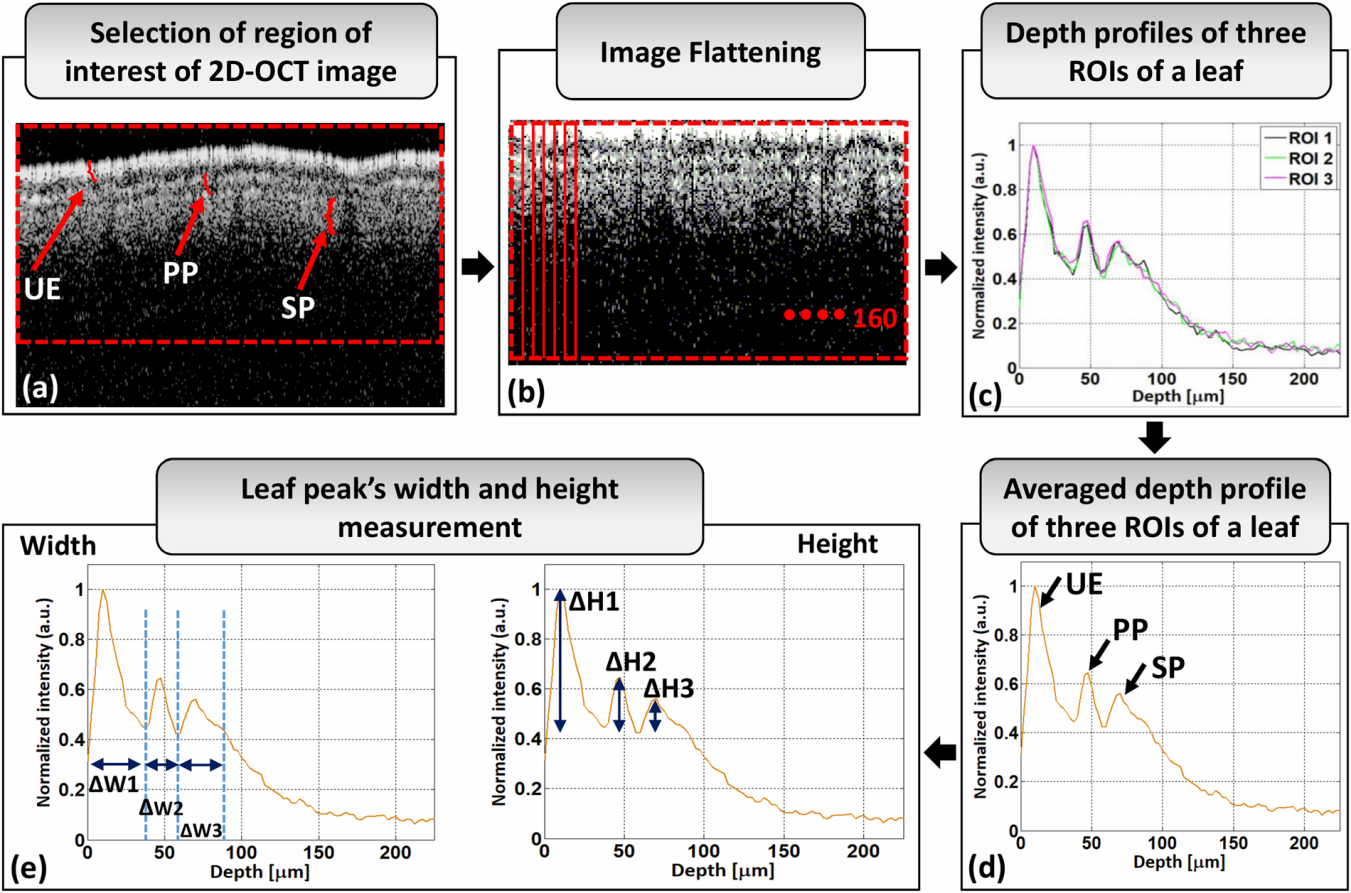
Width and height measurement algorithm of apple leaf layer intensity peaks. (**a**) 2D cross-sectional image of apple leaf with different layers. (**b**) 2D cross-sectional image after flattening. (**c**) Depth profiles of three regions of interest (ROIs) of a single leaf. (**d**) Average depth profile of three ROIs of a single leaf. (**e**) Width and height measurement of leaf layer intensity peaks. UE: upper epidermis, PP: palisade parenchyma, SP: spongy parenchyma.

The algorithm used for evaluating apple leaf layer intensity peak width and height measurements is shown in Fig. 5. Image acquisition, grayscale conversion, flattening, filtering, depth profiling, and normalization of 2D cross-sectional OCT images (Fig. 5(a–c)) of apple leaves were performed using the same approach described for the persimmon leaf thickness measurement algorithm. The depth intensity profiles of three ROIs were then averaged to obtain a more reliable depth intensity profile for the apple leaf, as shown in Fig. 5(d). Finally, the width and height of the apple leaf layer intensity peaks were measured to detect healthy, apparently-healthy, and infected leaves. The width and height measurement process of apple leaf layer intensity peaks is shown in Fig. 5(e), where ΔW and ΔH indicate the width and height of the intensity peak, respectively. The custom code described in this section was developed according to a previously reported method^58^.

### On-field qualitative inspection

**Fig. 6:**
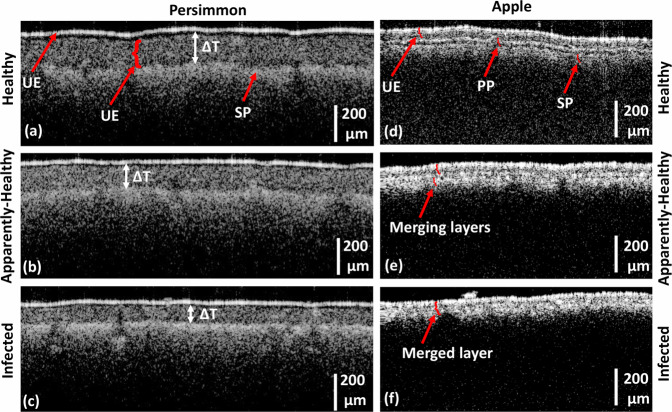
2D cross-sectional images of healthy, apparently-healthy, and infected persimmon and apple leaves. (**a–c**) 2D cross-sectional images of healthy, apparently-healthy, and infected persimmon leaves, respectively. (**d**–**f**) 2D cross-sectional images of healthy, apparently-healthy, and infected apple leaves, respectively. UE: upper epidermis, PP: palisade parenchyma, SP: spongy parenchyma.

OCT cross-sectional images of healthy, apparently-healthy, and infected persimmon and apple leaves are shown in Fig. 6. OCT cross-sectional images of healthy, apparently-healthy, and infected persimmon leaves were visualized with UE, PP, and SP layers (marked with red arrows) and are shown in Fig. 6(a–c), respectively. The thickness difference between the UE and SP layers of healthy, apparently-healthy, and infected persimmon leaves, indicated using white arrows, is clearly distinguishable in OCT images. Similarly, Fig. 6(d–f) show the OCT cross-sectional images of healthy, apparently-healthy, and infected apple leaves, respectively. The healthy apple leaf is visualized with distinguishable UE, PP, and SP layers, indicated by red arrows in Fig. 6(d). The apparently-healthy apple leaf is visualized with the UE layer and merged PP and SP layers, indicated by the red arrow in Fig. 6(e). Moreover, the distance between the UE layer and merged PP and SP layers of the apparently-healthy apple leaf is lower than that of the healthy apple leaf. In infected apple leaves, all three layers merged and appeared as a single layer, indicated by the red arrow in Fig. 6(f).

### Quantified thickness-based thresholding

**Fig. 7:**
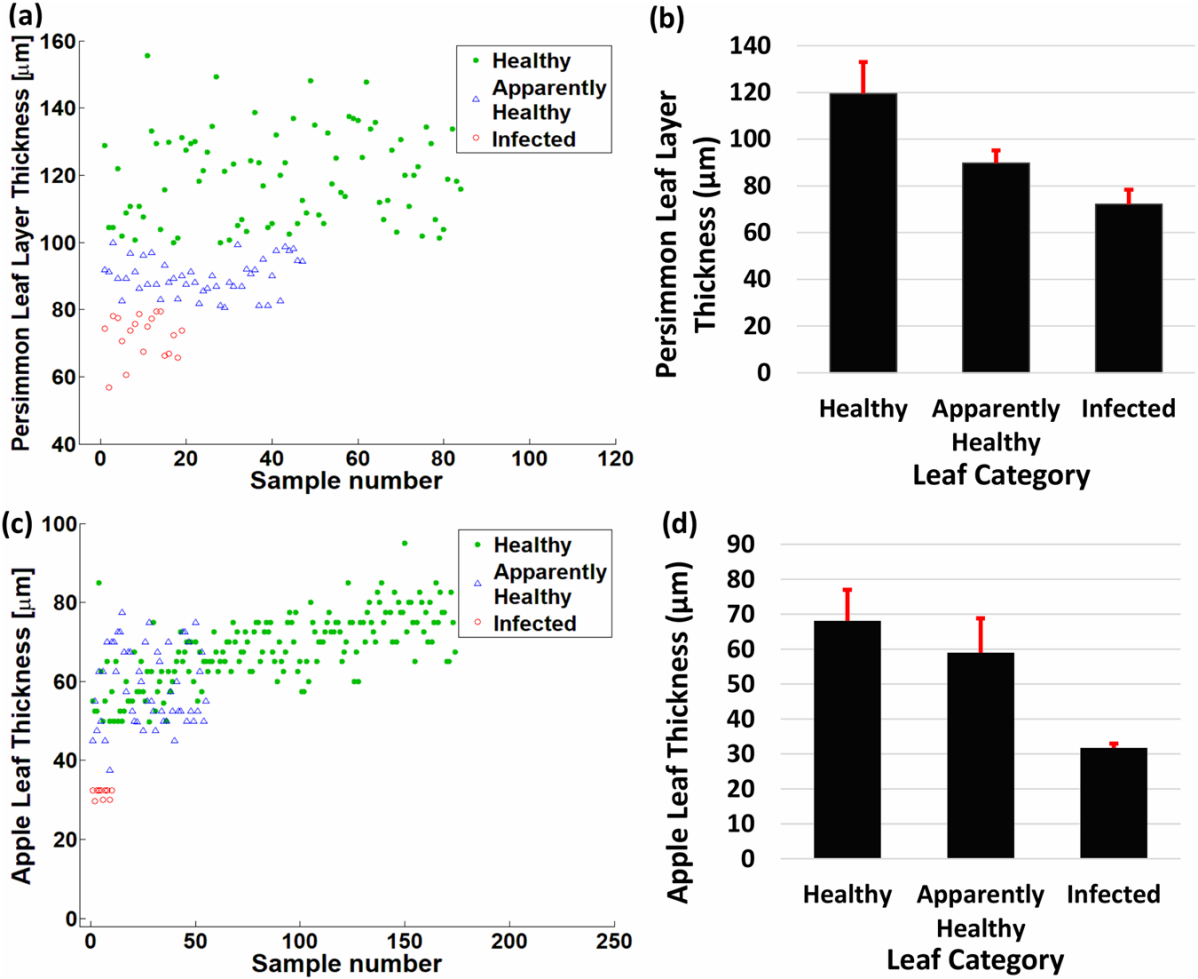
Persimmon and apple leaf layer thickness. (**a**) Scatter plot of the layer thickness of healthy, apparently-healthy, and infected persimmon leaves. (**b**) Comparison of layer thickness of persimmon leaves. (**c**) Scatter plot of the layer thickness of healthy, apparently-healthy, and infected apple leaves. (**b**) Comparison of layer thickness of apple leaves.

PP layer thickness differences between healthy, apparently-healthy, and infected specimens of persimmon leaf are shown in Fig. 7(a,b). In Fig. 7(a), the scatter plot presents the range of PP layer thickness of persimmon leaves, where it is visualized that the range of the PP layer thickness of healthy, apparently-healthy, and infected persimmon leaves is 100–160, 80–100, and >80 µm, respectively. Figure 7(b) shows the average thickness of the PP layers of healthy, apparently-healthy, and infected persimmon leaves with an average thickness of 119.5, 89.8, and 72.1 µm, respectively. The thickness range of healthy, apparently-healthy, and infected apple leaves is shown in Fig. 7(c) using a scatter plot, where the thickness range of most healthy apple leaves is 60–80 µm. Moreover, the overall range of thickness of apparently-healthy and infected apple leaves declined compared with that of healthy leaves. Figure 7(d) shows the average thicknesses of healthy, apparently-healthy, and infected apple leaves to be 68.1, 59, and 31.7 µm, respectively. Moreover, the scatter plots in Fig. 7(a,c) present the ratio of healthy, apparently-healthy, and infected samples in persimmon and apple fields, respectively, where leaf samples were selected randomly.

### Signal peak detection

**Fig. 8:**
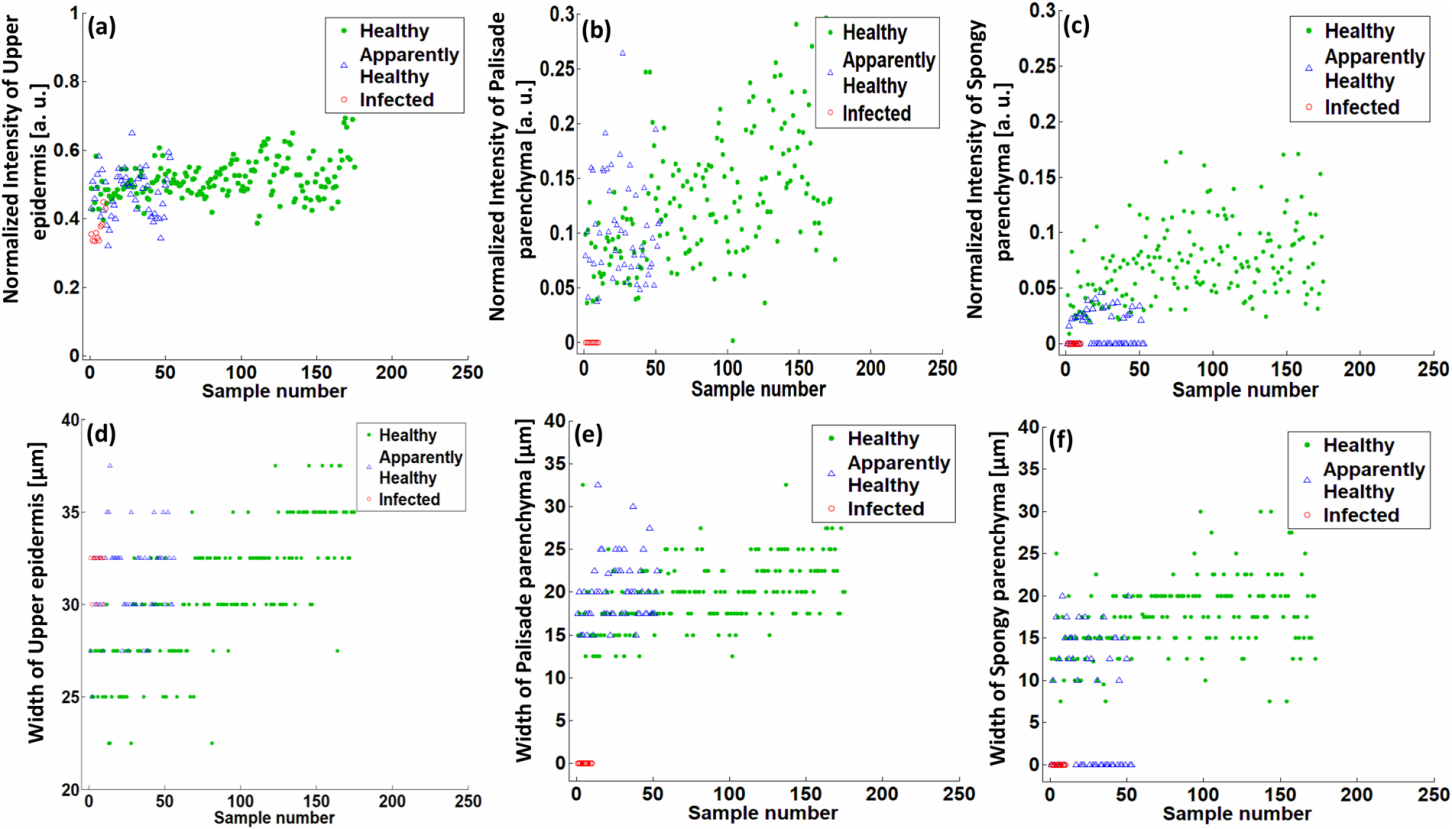
Scatter plot of upper epidermis (UE), palisade parenchyma (PP), and spongy parenchyma (SP) layer peak width and height of healthy, apparently-healthy, and infected apple leaf intensity. (**a**) Intensity peak height/normalized intensity of the UE layer peak of healthy, apparently-healthy, and infected apple leaves. (**b**) Intensity height of the PP layer peak of apple leaves. (**c**) Intensity height of the SP peak of apple leaves. (**d**) UE layer peak width of healthy, apparently-healthy, and infected apple leaves. (**e**) PP layer peak width of apple leaves. (**f**) SP layer peak width of apple leaves.

Figure 8 shows the UE, PP, and SP layer intensity peak height/normalized intensity and width of healthy, apparently-healthy, and infected apple leaves. The method for measuring intensity peak height and width is discussed in Section 2.3. UE, PP, and SP layer intensity peak heights of apple leaves are shown in Fig. 8(a–c), respectively. As seen in Fig. 8(a), UE layer peak heights of healthy and apparently-healthy apple leaves are approximately the same, with the scatter plot showing no significant difference between them; however, the UE layer peak of infected leaves has declined compared with that of healthy and apparently-healthy leaves. In Fig. 8(b), the scatter plot shows that the height of the PP layer peak of apparently-healthy apple leaves has declined compared with that of healthy leaf specimens. Notably, the PP layer peak was not present in infected apple leaves and the position of their height can be identified at zero level in the scatter plot. Figure 8(c) shows the gradual decline of the SP layer intensity peak height of apparently-healthy and infected apple leaves compared with that of healthy leaf specimens, where the SP layer intensity peak was not present in all infected leaf specimens, and most apparently-healthy specimens and the position of their height was seen at zero level in the scatter plot.

The scatter plots of the UE layer peak width of healthy, apparently-healthy, and infected apple leaves do not exhibit a significant difference, as shown in Fig. 8(d). The plotting of the PP layer peak width of infected leaf specimens is seen at the zero level of the scatter plot owing to its merging with the UE layer intensity peak, as shown in Fig. 8(e). Moreover, Fig. 8(e) reveals that the width of the PP layer intensity peak of healthy and apparently-healthy leaf specimens was nearly the same. The gradual decline of the intensity peak width of apparently-healthy and infected apple leaves compared with the healthy leaf specimens is plotted in Fig. 8(f). The SP layer peak merged with the PP layer peak in most apparently-healthy apple leaves. PP and SP layer peaks merged with the UE layer peak in infected apple leaves, and their plotting can be identified at the zero level of the scatter plot.

### Optical signal intensity assessments

**Fig. 9:**
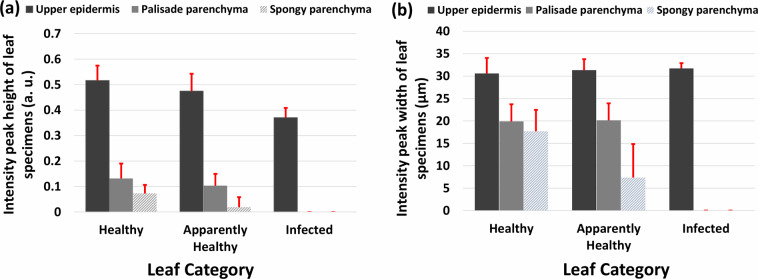
Comparison of average height and width of intensity peaks of healthy, apparently-healthy, and infected apple leaves. (**a**) Comparison of the intensity peak heights of healthy, apparently-healthy, and infected apple leaves. (**b**) Comparison of the intensity peak widths of healthy, apparently-healthy, and infected apple leaves.

A comparison of the average height and width of intensity peaks of healthy, apparently-healthy, and infected apple leaves is shown in Fig. 9. Figure 9(a) shows a comparison of the average height of intensity peaks. Intensities of the UE, PP, and SP layer peaks of apparently-healthy and infected apple leaves declined compared with healthy apple leaves. Notably, the PP and SP layer peaks disappeared in infected apple leaves due to merging with the UE layer peak. Figure 9(b) shows a comparison of the average width of intensity peaks, where not much difference between the UE layer intensity peaks of healthy, apparently-healthy, and infected apple leaves was observed. Moreover, the average width of the PP layer peaks of healthy and apparently-healthy is nearly the same. The average width of the SP layer peak of apparently-healthy leaves significantly declined compared with that of healthy leaves due to merging with the PP layer peak, as shown in Fig. 9(b). The PP and SP layer intensity peaks of infected apple leaves are not visible in Fig. 9(b) due to the merging of the PP and SP layer intensity peaks with the UE layer peak.

Our results revealed a decrease in PP layer thickness in apparently-healthy and infected persimmon leaves compared with healthy persimmon leaves. Moreover, the SP layer of apparently-healthy apple leaves merged with the PP layer. Similarly, the PP and SP layers merged with the UE layer in infected apple leaves; subsequently, a significant thickness difference was found in infected apple leaves. After detecting the significant thickness difference, a large-scale OCT image set was incorporated and averaged to set a reliable threshold for detecting healthy, apparently-healthy, and infected leaves. The average thicknesses of healthy, apparently-healthy, and infected apple leaves were 68.12, 58.95, and 31.72 µm, respectively; the average thicknesses of PP layers of healthy, apparently-healthy, and infected persimmon leaves were 119.46, 89.78, and 72.09 µm, respectively. In addition, the scatter plots (shown in Fig. 7) displayed the ratio and thickness variations of healthy, apparently-healthy, and infected leaves chosen randomly from persimmon and apple fields. The scatter plots (shown in Fig. 8) show variations in width and height of UE, PP, and SP layer intensity peaks of apple leaves.

The initial symptoms of plant diseases appear mostly on the leaf subsurface, which is a complex organ composed primarily of palisade parenchyma (PP) and spongy parenchyma (SP), crossed by vascular tissue, and enclosed by two epidermises^11^. In this study, a developed depth profile algorithm was applied to cross-sectional OCT images of apple and persimmon leaves to quantitatively evaluate the inner structure of PP and SP layers, which are often seen in all types of leaves. As with all forms of leaf diseases, the PP and SP are affected, and their abnormalities can be identified by setting a threshold using the proposed method of this study, which allows this approach to work with different genotypes. Minimum Information About a Plant Phenotyping Experiment (MIAPPE) standard checklist has been given in the supplementary file.

## Data Records

In this study, all OCT images of persimmon and apple leaves were acquired in-field and nondestructively using a backpack-type wearable OCT system from the persimmon and apple fields located in Daegu, South Korea. Obtained OCT images were employed to set a threshold for the pre-identification of palisade parenchyma (PP) and spongy parenchyma (SP) layer abnormalities of persimmon and apple leaves, which can subsequently reveal the symptoms of a diseased tree. However, three leaves were randomly chosen and imaged from each apple and persimmon tree. A total of 600 OCT images from 150 leaves (4 from each leaf), and 723 OCT images from 241 apple leaves (3 from each leaf) were used to set a threshold for the pre-identification of leaf abnormalities. Since, leaf layer abnormality is one of the major symptoms of plant disease, the proposed methodology of evaluating PP and SP leaf layer abnormalities can be used to pre-identify the presence of disease in a tree. A zipped file, containing the cross-sectional OCT images (in.tif format) of apple and persimmon leaves has been uploaded to Figshare^63^. The measured persimmon and apple leaf layer thicknesses; widths and heights of normalized intensity peaks of UE, PP, and SP layers are listed in Tables 2 to 9.

**Table 2:** Persimmon leaves layer thicknesses.

Healthy															
Leaf#	Thickness (µm)	Leaf#	Thickness (µm)	Leaf#	Thickness (µm)	Leaf#	Thickness (µm)	Leaf#	Thickness (µm)	Leaf#	Thickness (µm)	Leaf#	Thickness (µm)	Leaf#	Thickness (µm)
1	128.75	12	133.125	23	118.125	34	103.25	45	136.875	56	114.875	67	112.5	78	106.875
2	104.375	13	129.375	24	121.25	35	124.375	46	105.625	57	113.75	68	127.5	79	101.25
3	104.375	14	103.75	25	126.875	36	138.75	47	112.5	58	137.5	69	103.125	80	103.75
4	121.875	15	115.625	26	134.5	37	123.75	48	108.75	59	136.875	70	130.625	81	118.75
5	101.875	16	129.875	27	149.375	38	116.875	49	148.125	60	136.25	71	120	82	133.75
6	108.75	17	100	28	100	39	104.375	50	135	61	125.25	72	110.625	83	118.125
7	110.625	18	101.25	29	121.125	40	105.625	51	108.125	62	147.625	73	119.875	84	115.8
8	100.625	19	131.25	30	100.625	41	131.875	52	105.625	63	133.75	74	122.5		
9	110.625	20	127.5	31	123.25	42	120	53	132.5	64	135.625	75	101.875		
10	107.5	21	129.375	32	105	43	123.75	54	117.5	65	111.875	76	134.375		
11	155.625	22	130	33	106.875	44	102.5	55	125	66	106.875	77	129.375		
**Apparently-Healthy**															
**Leaf#**	**Thickness (µm)**	**Leaf#**	**Thickness (µm)**	**Leaf#**	**Thickness (µm)**	**Leaf#**	**Thickness (µm)**	**Leaf#**	**Thickness (µm)**	**Leaf#**	**Thickness (µm)**	**Leaf#**	**Thickness (µm)**	**Leaf#**	**Thickness (µm)**
85	91.875	91	96.75	97	87.5	103	90	109	86.25	115	87	121	81.25	127	98.75
86	91.25	92	91.25	98	83	104	87.5	110	90	116	99.375	122	95	128	97.5
87	99.875	93	86.375	99	93.125	105	91.25	111	86.875	117	86.875	123	81.25	129	98.125
88	89.375	94	96.25	100	88.125	106	88.125	112	81.25	118	92	124	90	130	94.5
89	82.5	95	87.5	101	89.375	107	81.875	113	80.625	119	90.625	125	97.5	131	94.375
90	89.375	96	96.875	102	83.125	108	85.625	114	88.125	120	91.875	126	82.5		
**Infected**															
**Leaf#**	**Thickness (µm)**	**Leaf#**	**Thickness (µm)**	**Leaf#**	**Thickness (µm)**	**Leaf#**	**Thickness (µm)**	**Leaf#**	**Thickness (µm)**	**Leaf#**	**Thickness (µm)**	**Leaf#**	**Thickness (µm)**	**Leaf#**	**Thickness (µm)**
132	74.375	135	77.5	138	73.75	141	67.5	144	79.375	147	66.875	150	73.75		
133	56.875	136	70.625	139	75.625	142	75	145	79.375	148	72.375				
134	78.125	137	60.625	140	78.75	143	77.375	146	66.25	149	65.625

**Table 3:** Apple leaves layer thicknesses.

Healthy															
Leaf #	Thickness (µm)	Leaf #	Thickness (µm)	Leaf #	Thickness (µm)	Leaf #	Thickness (µm)	Leaf #	Thickness (µm)	Leaf #	Thickness (µm)	Leaf #	Thickness (µm)	Leaf #	Thickness (µm)
1	55	23	57.5	45	67.5	67	62.5	89	60	111	70	133	80	155	65
2	52.5	24	65	46	70	68	75	90	70	112	72.5	134	75	156	75
3	52.5	25	57.5	47	62.5	69	70	91	65	113	75	135	72.5	157	82.5
4	85	26	55	48	70	70	72.5	92	62.5	114	77.5	136	70	158	80
5	62.5	27	62.5	49	60	71	65	93	67.5	115	72.5	137	82.5	159	72.5
6	50	28	49.7	50	70	72	67.5	94	75	116	72.5	138	80	160	77.5
7	55	29	62.5	51	55	73	67.5	95	77.5	117	75	139	85	161	70
8	65	30	75	52	67.5	74	72.5	96	75	118	75	140	75	162	80
9	50	31	52.5	53	57.5	75	70	97	70	119	72.5	141	77.5	163	70
10	57.5	32	57.5	54	65	76	62.5	98	77.5	120	65	142	70	164	75
11	50	33	60	55	65	77	62.5	99	62.5	121	67.5	143	77.5	165	85
12	65	34	62.5	56	62.5	78	72.5	100	65	122	70	144	70	166	75
13	50	35	54.5	57	65	79	75	101	57.5	123	85	145	82.5	167	82.5
14	52.5	36	50	58	65	80	75	102	57.5	124	75	146	70	168	77.5
15	50	37	62.5	59	72.5	81	65	103	65	125	75	147	80	169	77.5
16	52.5	38	57.5	60	67.5	82	67.5	104	60	126	60	148	77.5	170	65
17	60	39	60	61	62.5	83	72.5	105	80	127	67.5	149	77.5	171	65
18	55	40	62.5	62	65	84	75	106	75	128	60	150	95	172	82.5
19	55	41	65	63	67.5	85	72.5	107	72.5	129	75	151	72.5	173	75
20	55	42	67.5	64	65	86	75	108	65	130	75	152	80	174	67.5
21	67.5	43	72.5	65	70	87	67.5	109	70	131	70	153	80	175	70
22	57.5	44	65	66	70	88	65	110	70	132	77.5	154	75		
**Apparently-Healthy**															
**Leaf #**	**Thickness (µm)**	**Leaf #**	**Thickness (µm)**	**Leaf #**	**Thickness (µm)**	**Leaf #**	**Thickness (µm)**	**Leaf #**	**Thickness (µm)**	**Leaf #**	**Thickness (µm)**	**Leaf #**	**Thickness (µm)**	**Leaf #**	**Thickness (µm)**
176	45	183	70	190	77.5	197	49.7	204	55	211	50	218	52.5	225	75
177	55	184	37.5	191	67.5	198	62.5	205	52.5	212	70	219	72.5	226	52.5
178	47.5	185	70	192	57.5	199	60	206	47.5	213	57.5	220	72.5	227	62.5
179	62.5	186	70	193	67.5	200	47.5	207	67.5	214	52.5	221	50	228	67.5
180	50	187	62.5	194	67.5	201	70	208	65	215	45	222	70		
181	62.5	188	72.5	195	52.5	202	55	209	52.5	216	60	223	52.5		
182	45	189	72.5	196	50	203	75	210	50	217	52.5	224	50		
**Infected**															
**Leaf #**	**Thickness (µm)**	**Leaf #**	**Thickness (µm)**	**Leaf #**	**Thickness (µm)**	**Leaf #**	**Thickness (µm)**	**Leaf #**	**Thickness (µm)**	**Leaf #**	**Thickness (µm)**	**Leaf #**	**Thickness (µm)**	**Leaf #**	**Thickness (µm)**
232	32.5	234	32.5	236	32.5	238	32.5	240	30						
233	29.7	235	32.5	237	30	239	32.5	241	32.5

**Table 4:** Apple leaves upper epidermis layer peak height.

Healthy (1^st^ Peak)															
Leaf#	Height (a. u.)	Leaf#	Height (a. u.)	Leaf#	Height (a. u.)	Leaf#	Height (a. u.)	Leaf#	Height (a. u.)	Leaf#	Height (a. u.)	Leaf#	Height (a. u.)	Leaf#	Height (a. u.)
1	0.4893	23	0.4938	45	0.5217	67	0.476	89	0.5488	111	0.3862	133	0.6169	155	0.4579
2	0.4265	24	0.5456	46	0.6066	68	0.5152	90	0.5152	112	0.407	134	0.6492	156	0.4921
3	0.4467	25	0.5043	47	0.546	69	0.5174	91	0.4685	113	0.5967	135	0.4485	157	0.5138
4	0.5817	26	0.4952	48	0.5841	70	0.5367	92	0.4818	114	0.6119	136	0.4589	158	0.5073
5	0.4931	27	0.5136	49	0.5082	71	0.469	93	0.5601	115	0.6238	137	0.527	159	0.5716
6	0.4287	28	0.4974	50	0.5296	72	0.5492	94	0.5712	116	0.616	138	0.5283	160	0.431
7	0.4709	29	0.493	51	0.482	73	0.4797	95	0.5875	117	0.5746	139	0.5286	161	0.4907
8	0.5095	30	0.5471	52	0.5391	74	0.4723	96	0.526	118	0.6332	140	0.4958	162	0.5225
9	0.3962	31	0.4728	53	0.477	75	0.4894	97	0.5723	119	0.5068	141	0.4815	163	0.4945
10	0.4838	32	0.4342	54	0.5035	76	0.4592	98	0.5035	120	0.4701	142	0.4342	164	0.4492
11	0.4447	33	0.525	55	0.5139	77	0.4636	99	0.5258	121	0.4809	143	0.46	165	0.5487
12	0.4839	34	0.5389	56	0.5293	78	0.506	100	0.4828	122	0.528	144	0.5051	166	0.5972
13	0.466	35	0.4766	57	0.5036	79	0.4913	101	0.4945	123	0.5571	145	0.5762	167	0.5704
14	0.4558	36	0.4146	58	0.5057	80	0.5142	102	0.4802	124	0.5828	146	0.4537	168	0.6807
15	0.4642	37	0.4883	59	0.4953	81	0.4181	103	0.5239	125	0.5832	147	0.4254	169	0.6925
16	0.4737	38	0.4608	60	0.4969	82	0.4957	104	0.4874	126	0.4364	148	0.5977	170	0.6677
17	0.4716	39	0.4562	61	0.4602	83	0.5195	105	0.5647	127	0.5378	149	0.5072	171	0.5791
18	0.4853	40	0.4803	62	0.5077	84	0.5351	106	0.5064	128	0.5466	150	0.5289	172	0.5738
19	0.4645	41	0.5051	63	0.5333	85	0.5343	107	0.5411	129	0.6246	151	0.463	173	0.7235
20	0.4625	42	0.5045	64	0.5249	86	0.5305	108	0.5427	130	0.5466	152	0.4537	174	0.6893
21	0.5442	43	0.5846	65	0.4829	87	0.5446	109	0.5396	131	0.5772	153	0.4933	175	0.5511
22	0.4863	44	0.513	66	0.5496	88	0.4846	110	0.5649	132	0.6221	154	0.5448		
**Apparently-Healthy (1**^**st**^ **Peak)**															
**Leaf#**	**Height (a. u.)**	**Leaf#**	**Height (a. u.)**	**Leaf#**	**Height (a. u.)**	**Leaf#**	**Height (a. u.)**	**Leaf#**	**Height (a. u.)**	**Leaf#**	**Height (a. u.)**	**Leaf#**	**Height (a. u.)**	**Leaf#**	**Height (a. u.)**
176	0.4308	183	0.5421	190	0.4503	197	0.501	204	0.5008	211	0.5226	218	0.4159	225	0.4986
177	0.5092	184	0.4254	191	0.4392	198	0.5476	205	0.4555	212	0.5536	219	0.4007	226	0.5146
178	0.4586	185	0.5066	192	0.4001	199	0.5195	206	0.4291	213	0.4953	220	0.4769	227	0.5941
179	0.4883	186	0.3818	193	0.5221	200	0.5035	207	0.5174	214	0.4258	221	0.4007	228	0.5785
180	0.5293	187	0.322	194	0.5472	201	0.4932	208	0.5487	215	0.4057	222	0.343		
181	0.5824	188	0.367	195	0.5222	202	0.4708	209	0.5239	216	0.4057	223	0.4425		
182	0.4057	189	0.4073	196	0.5113	203	0.6503	210	0.4889	217	0.3908	224	0.4041		
**Infected (1**^**st**^ **Peak)**															
**Leaf#**	**Height (a. u.)**	**Leaf#**	**Height (a. u.)**	**Leaf#**	**Height (a. u.)**	**Leaf#**	**Height (a. u.)**	**Leaf#**	**Height (a. u.)**	**Leaf#**	**Height (a. u.)**	**Leaf#**	**Height (a. u.)**	**Leaf#**	**Height (a. u.)**
232	0.3545	234	0.3341	236	0.3443	238	0.3784	240	0.4488						
233	0.3356	235	0.3591	237	0.3366	239	0.3832	241	0.4305

**Table 5:** Apple leaves palisade parenchyma layer peak height.

Healthy (2^nd^ Peak)															
Leaf#	Height (a. u.)	Leaf#	Height (a. u.)	Leaf#	Height (a. u.)	Leaf#	Height (a. u.)	Leaf#	Height (a. u.)	Leaf#	Height (a. u.)	Leaf#	Height (a. u.)	Leaf#	Height (a. u.)
1	0.0989	23	0.0546	45	0.1363	67	0.1246	89	0.1611	111	0.0848	133	0.2433	155	0.1363
2	0.0363	24	0.0965	46	0.2474	68	0.1036	90	0.1174	112	0.0608	134	0.2561	156	0.1452
3	0.103	25	0.0837	47	0.1524	69	0.143	91	0.0584	113	0.1945	135	0.0925	157	0.2173
4	0.1284	26	0.0779	48	0.2013	70	0.0958	92	0.1102	114	0.0869	136	0.1545	158	0.1824
5	0.0907	27	0.1143	49	0.1798	71	0.0816	93	0.1869	115	0.2204	137	0.2445	159	0.2711
6	0.0379	28	0.0544	50	0.1316	72	0.1475	94	0.2006	116	0.2374	138	0.1956	160	0.1323
7	0.0605	29	0.1227	51	0.1207	73	0.0835	95	0.2131	117	0.1918	139	0.1728	161	0.109
8	0.0876	30	0.1126	52	0.1413	74	0.0845	96	0.1848	118	0.2248	140	0.2005	162	0.0845
9	0.04	31	0.0587	53	0.098	75	0.1405	97	0.1722	119	0.1978	141	0.1415	163	0.1298
10	0.109	32	0.0529	54	0.1119	76	0.068	98	0.1519	120	0.097	142	0.1457	164	0.1091
11	0.0639	33	0.1126	55	0.1654	77	0.1142	99	0.1143	121	0.1262	143	0.176	165	0.1
12	0.0603	34	0.1089	56	0.1963	78	0.1528	100	0.0925	122	0.1054	144	0.148	166	0.2489
13	0.0541	35	0.0821	57	0.1163	79	0.0902	101	0.0831	123	0.1357	145	0.229	167	0.127
14	0.063	36	0.0398	58	0.0995	80	0.1627	102	0.063	124	0.1495	146	0.2074	168	0.2131
15	0.0855	37	0.0593	59	0.0765	81	0.0718	103	0.159	125	0.1283	147	0.1614	169	0.2968
16	0.096	38	0.0411	60	0.1278	82	0.0939	104	0.0017	126	0.0361	148	0.2908	170	0.22307
17	0.0929	39	0.0691	61	0.0638	83	0.1509	105	0.1285	127	0.1505	149	0.178	171	0.1288
18	0.0716	40	0.0678	62	0.1529	84	0.1433	106	0.0828	128	0.1198	150	0.1932	172	0.1312
19	0.0788	41	0.0844	63	0.1318	85	0.1575	107	0.1464	129	0.221	151	0.1014	173	0.2512
20	0.0942	42	0.0916	64	0.1631	86	0.1305	108	0.12	130	0.1198	152	0.1513	174	0.2335
21	0.1373	43	0.2474	65	0.063	87	0.1622	109	0.1326	131	0.178	153	0.1922	175	0.0761
22	0.0859	44	0.0757	66	0.1576	88	0.1165	110	0.1035	132	0.2127	154	0.2294		
**Apparently-Healthy (2**^**nd**^ **Peak)**															
**Leaf#**	**Height (a. u.)**	**Leaf#**	**Height (a. u.)**	**Leaf#**	**Height (a. u.)**	**Leaf#**	**Height (a. u.)**	**Leaf#**	**Height (a. u.)**	**Leaf#**	**Height (a. u.)**	**Leaf#**	**Height (a. u.)**	**Leaf#**	**Height (a. u.)**
176	0.0795	183	0.1083	190	0.1915	197	0.0841	204	0.0998	211	0.1346	218	0.0529	225	0.195
177	0.1015	184	0.0372	191	0.1588	198	0.1073	205	0.0551	212	0.0528	219	0.1071	226	0.0881
178	0.0417	185	0.0401	192	0.1012	199	0.068	206	0.1401	213	0.0698	220	0.0623	227	0.1088
179	0.0752	186	0.1003	193	0.1385	200	0.1721	207	0.1623	214	0.0482	221	0.0691	228	0.1109
180	0.1601	187	0.0729	194	0.1625	201	0.1025	208	0.0689	215	0.0872	222	0.0718		
181	0.1576	188	0.1588	195	0.0589	202	0.2643	209	0.0863	216	0.0802	223	0.0953		
182	0.0719	189	0.157	196	0.1117	203	0.0715	210	0.0801	217	0.1416	224	0.0521		
**Infected (2**^**nd**^ **Peak)**															
**Leaf#**	**Height (a. u.)**	**Leaf#**	**Height (a. u.)**	**Leaf#**	**Height (a. u.)**	**Leaf#**	**Height (a. u.)**	**Leaf#**	**Height (a. u.)**	**Leaf#**	**Height (a. u.)**	**Leaf#**	**Height (a. u.)**	**Leaf#**	**Height (a. u.)**
232	0	234		236	0	238	0	240	0						
233	0	235		237	0	239	0	241	0

**Table 6:** Apple leaves spongy parenchyma layer peak height.

Healthy (3^rd^ Peak)															
Leaf#	Height (a. u.)	Leaf#	Height (a. u.)	Leaf#	Height (a. u.)	Leaf#	Height (a. u.)	Leaf#	Height (a. u.)	Leaf#	Height (a. u.)	Leaf#	Height (a. u.)	Leaf#	Height (a. u.)
1	0.0439	23	0.0494	45	0.04	67	0.0954	89	0.0553	111	0.0504	133	0.1416	155	0.0903
2	0.0088	24	0.0324	46	0.0763	68	0.1642	90	0.065	112	0.0545	134	0.0831	156	0.102
3	0.0344	25	0.0771	47	0.0538	69	0.1024	91	0.0534	113	0.1152	135	0.047	157	0.1092
4	0.083	26	0.0465	48	0.0727	70	0.0754	92	0.0858	114	0.1397	136	0.0245	158	0.1713
5	0.0333	27	0.0681	49	0.08	71	0.0308	93	0.055	115	0.0774	137	0.0929	159	0.0936
6	0.0255	28	0.0554	50	0.1162	72	0.1137	94	0.161	116	0.0647	138	0.0991	160	0.1323
7	0.0228	29	0.033	51	0.0832	73	0.0594	95	0.0738	117	0.0523	139	0.0932	161	0.0961
8	0.0651	30	0.0743	52	0.0681	74	0.0541	96	0.1368	118	0.0989	140	0.0789	162	0.0555
9	0.0287	31	0.0569	53	0.0302	75	0.069	97	0.1192	119	0.0711	141	0.0684	163	0.0361
10	0.0514	32	0.079	54	0.0965	76	0.0501	98	0.1382	120	0.0686	142	0.0537	164	0.0445
11	0.0277	33	0.0653	55	0.1133	77	0.0814	99	0.0475	121	0.0368	143	0.0462	165	0.069
12	0.0932	34	0.0491	56	0.0784	78	0.1723	100	0.0756	122	0.0378	144	0.064	166	0.1173
13	0.041	35	0.0235	57	0.0714	79	0.0753	101	0.0524	123	0.0737	145	0.0873	167	0.0493
14	0.0245	36	0.0217	58	0.0338	80	0.1199	102	0.0465	124	0.0693	146	0.0579	168	0.0722
15	0.0534	37	0.0687	59	0.0652	81	0.031	103	0.116	125	0.0413	147	0.0687	169	0.0905
16	0.0206	38	0.043	60	0.078	82	0.0507	104	0.0713	126	0.0422	148	0.1704	170	0.1166
17	0.0381	39	0.0755	61	0.0424	83	0.0735	105	0.0871	127	0.0313	149	0.118	171	0.0315
18	0.0531	40	0.0343	62	0.085	84	0.1008	106	0.0827	128	0.055	150	0.1294	172	0.0449
19	0.0356	41	0.0441	63	0.0879	85	0.0795	107	0.1217	129	0.0771	151	0.047	173	0.1528
20	0.0484	42	0.0719	64	0.0776	86	0.1185	108	0.1159	130	0.067	152	0.0644	174	0.0965
21	0.1057	43	0.1249	65	0.0582	87	0.0892	109	0.1122	131	0.0653	153	0.0757	175	0.0559
22	0.0359	44	0.028	66	0.1117	88	0.0599	110	0.0881	132	0.0998	154	0.089		
**Apparently-Healthy (3**^**rd**^ **Peak)**															
**Leaf#**	**Height (a. u.)**	**Leaf#**	**Height (a. u.)**	**Leaf#**	**Height (a. u.)**	**Leaf#**	**Height (a. u.)**	**Leaf#**	**Height (a. u.)**	**Leaf#**	**Height (a. u.)**	**Leaf#**	**Height (a. u.)**	**Leaf#**	**Height (a. u.)**
176	0	183	0.0246	190	0.039	197	0	204	0	211	0	218	0.0267	225	0.0341
177	0.0162	184	0	191	0.0199	198	0	205	0	212	0	219	0	226	0.0214
178	0	185	0.027	192	0	199	0.0463	206	0.0242	213	0	220	0.0337	227	0
179	0.0222	186	0.0214	193	0.0316	200	0.0322	207	0.0369	214	0.0232	221	0	228	0
180	0	187	0.0272	194	0	201	0	208	0	215	0	222	0		
181	0.024	188	0.0235	195	0.0408	202	0.0333	209	0	216	0	223	0.279		
182	0	189	0.0307	196	0	203	0	210	0.0372	217	0.0257	224	0		
**Infected (3**^**rd**^ **Peak Height)**															
**Leaf#**	**Height (a. u.)**	**Leaf#**	**Height (a. u.)**	**Leaf#**	**Height (a. u.)**	**Leaf#**	**Height (a. u.)**	**Leaf#**	**Height (a. u.)**	**Leaf#**	**Height (a. u.)**	**Leaf#**	**Height (a. u.)**	**Leaf#**	**Height (a. u.)**
232	0	234	0	236	0	238	0	240	0						
233	0	235	0	237	0	239	0	241	0

**Table 7:** Apple leaves upper epidermis layer peak width.

Healthy (1^st^ Peak)															
Leaf#	Width (µm)	Leaf#	Width (µm)	Leaf#	Width (µm)	Leaf#	Width (µm)	Leaf#	Width (µm)	Leaf#	Width (µm)	Leaf#	Width (µm)	Leaf#	Width (µm)
1	27.5	23	25	45	32.5	67	25	89	30	111	32.5	133	35	155	35
2	25	24	27.5	46	30	68	35	90	32.5	112	32.5	134	32.5	156	35
3	25	25	25	47	27.5	69	25	91	30	113	32.5	135	35	157	32.5
4	27.5	26	27.5	48	30	70	32.5	92	27.5	114	32.5	136	30	158	32.5
5	27.5	27	27.5	49	25	71	30	93	32.5	115	32.5	137	32.5	159	35
6	25	28	22.5	50	32.5	72	32.5	94	30	116	32.5	138	35	160	37.5
7	27.5	29	30	51	25	73	32.5	95	35	117	30	139	35	161	35
8	27.5	30	32.5	52	30	74	32.5	96	30	118	32.5	140	32.5	162	32.5
9	25	31	30	53	27.5	75	32.5	97	30	119	32.5	141	35	163	35
10	27.5	32	27.5	54	27.5	76	30	98	30	120	30	142	35	164	27.5
11	25	33	27.5	55	27.5	77	30	99	32.5	121	30	143	35	165	37.5
12	30	34	27.5	56	27.5	78	32.5	100	32.5	122	30	144	35	166	37.5
13	22.5	35	30	57	27.5	79	30	101	30	123	37.5	145	37.5	167	32.5
14	22.5	36	25	58	25	80	32.5	102	30	124	32.5	146	30	168	35
15	25	37	30	59	27.5	81	22.5	103	30	125	35	147	30	169	35
16	27.5	38	27.5	60	27.5	82	27.5	104	30	126	30	148	35	170	35
17	27.5	39	27.5	61	27.5	83	32.5	105	35	127	30	149	35	171	32.5
18	27.5	40	27.5	62	30	84	32.5	106	32.5	128	30	150	35	172	32.5
19	27.5	41	32.5	63	30	85	30	107	32.5	129	32.5	151	32.5	173	35
20	25	42	30	64	27.5	86	32.5	108	32.5	130	30	152	35	174	35
21	25	43	30	65	27.5	87	30	109	30	131	35	153	35	175	35
22	25	44	30	66	30	88	30	110	32.5	132	32.5	154	37.5		
**Apparently-Healthy (1**^**st**^ **Peak)**															
**Leaf#**	**Width (µm)**	**Leaf#**	**Width (µm)**	**Leaf#**	**Width (µm)**	**Leaf#**	**Width (µm)**	**Leaf#**	**Width (µm)**	**Leaf#**	**Width (µm)**	**Leaf#**	**Width (µm)**	**Leaf#**	**Width (µm)**
176	27.5	183	32.5	190	32.5	197	27.5	204	30	211	27.5	218	35	225	30
177	25	184	27.5	191	32.5	198	30	205	30	212	32.5	219	30	226	32.5
178	32.5	185	30	192	32.5	199	30	206	30	213	27.5	220	32.5	227	35
179	30	186	32.5	193	32.5	200	30	207	32.5	214	27.5	221	32.5	228	32.5
180	30	187	35	194	32.5	201	27.5	208	32.5	215	32.5	222	32.5		
181	32.5	188	35	195	32.5	202	30	209	32.5	216	30	223	32.5		
182	30	189	37.5	196	32.5	203	35	210	30	217	30	224	35		
**Infected (1**^**st**^ **Peak)**															
**Leaf#**	**Width (µm)**	**Leaf#**	**Width (µm)**	**Leaf#**	**Width (µm)**	**Leaf#**	**Width (µm)**	**Leaf#**	**Width (µm)**	**Leaf#**	**Width (µm)**	**Leaf#**	**Width (µm)**	**Leaf#**	**Width (µm)**
232	32.5	234	32.5	236	32.5	238	32.5	240	30						
233	29.7	235	32.5	237	30	239	32.5	241	32.5

**Table 8:** Apple leaves palisade parenchyma layer peak width.

Healthy (2^nd^ Peak)															
Leaf#	Width (µm)	Leaf#	Width (µm)	Leaf#	Width (µm)	Leaf#	Width (µm)	Leaf#	Width (µm)	Leaf#	Width (µm)	Leaf#	Width (µm)	Leaf#	Width (µm)
1	15	23	20	45	22.5	67	20	89	17.5	111	17.5	133	22.5	155	20
2	17.5	24	22.5	46	22.5	68	20	90	17.5	112	20	134	25	156	22.5
3	15	25	15	47	20	69	25	91	20	113	20	135	20	157	22.5
4	32.5	26	12.5	48	20	70	20	92	17.5	114	25	136	25	158	22.5
5	17.5	27	15	49	17.5	71	20	93	15	115	20	137	32.5	159	17.5
6	12.5	28	15	50	17.5	72	15	94	20	116	20	138	25	160	22.5
7	20	29	17.5	51	15	73	17.5	95	22.5	117	25	139	20	161	25
8	20	30	20	52	20	74	20	96	22.5	118	22.5	140	22.5	162	22.5
9	15	31	12.5	53	17.5	75	17.5	97	20	119	25	141	22.5	163	27.5
10	15	32	15	54	17.5	76	15	98	17.5	120	17.5	142	17.5	164	27.5
11	12.5	33	17.5	55	22.5	77	20	99	17.5	121	22.5	143	17.5	165	25
12	15	34	17.5	56	20	78	20	100	15	122	22.5	144	20	166	25
13	12.5	35	15	57	17.5	79	25	101	17.5	123	22.5	145	25	167	27.5
14	15	36	17.5	58	25	80	20	102	12.5	124	20	146	22.5	168	22.5
15	12.5	37	17.5	59	25	81	27.5	103	17.5	125	22.5	147	20	169	25
16	15	38	15	60	22.2	82	25	104	15	126	15	148	25	170	22.5
17	17.5	39	17.5	61	17.5	83	20	105	17.5	127	25	149	20	171	17.5
18	15	40	20	62	17.5	84	22.5	106	20	128	17.5	150	20	172	17.5
19	17.5	41	12.5	63	20	85	22.5	107	17.5	129	20	151	17.5	173	27.5
20	20	42	20	64	22.5	86	22.5	108	17.5	130	22.5	152	17.5	174	20
21	25	43	22.5	65	25	87	17.5	109	20	131	20	153	22.5	175	20
22	17.5	44	22.5	66	20	88	17.5	110	20	132	25	154	25		
**Apparently-Healthy (2**^**nd**^ **Peak)**															
**Leaf#**	**Width (µm)**	**Leaf#**	**Width (µm)**	**Leaf#**	**Width (µm)**	**Leaf#**	**Width (µm)**	**Leaf#**	**Width (µm)**	**Leaf#**	**Width (µm)**	**Leaf#**	**Width (µm)**	**Leaf#**	**Width (µm)**
176	17.5	183	17.5	190	20	197	15	204	22.5	211	20	218	20	225	17.5
177	20	184	17.5	191	25	198	17.5	205	17.5	212	30	219	25	226	17.5
178	15	185	20	192	25	199	17.5	206	25	213	20	220	17.5	227	20
179	15	186	15	193	17.5	200	22.5	207	17.5	214	15	221	17.5	228	22.5
180	20	187	22.5	194	20	201	25	208	20	215	17.5	222	20		
181	17.5	188	20	195	17.5	202	22.5	209	20	216	17.5	223	27.5		
182	15	189	32.5	196	22.2	203	25	210	22.5	217	22.5	224	17.5		
**Infected (2**^**nd**^ **Peak)**															
**Leaf#**	**Width (µm)**	**Leaf#**	**Width (µm)**	**Leaf#**	**Width (µm)**	**Leaf#**	**Width (µm)**	**Leaf#**	**Width (µm)**	**Leaf#**	**Width (µm)**	**Leaf#**	**Width (µm)**	**Leaf#**	**Width (µm)**
232	0	234	0	236	0	238	0	240	0						
233	0	235	0	237	0	239	0	241	0

**Table 9:** Apple leaves spongy parenchyma layer peak width.

Healthy (3^rd^ Peak)															
Leaf#	Width (µm)	Leaf#	Width (µm)	Leaf#	Width (µm)	Leaf#	Width (µm)	Leaf#	Width (µm)	Leaf#	Width (µm)	Leaf#	Width (µm)	Leaf#	Width (µm)
1	12.5	23	12.5	45	12.5	67	17.5	89	12.5	111	20	133	17.5	155	20
2	10	24	15	46	17.5	68	20	90	20	112	20	134	15	156	27.5
3	12.5	25	17.5	47	15	69	20	91	15	113	22.5	135	17.5	157	27.5
4	25	26	15	48	20	70	20	92	17.5	114	20	136	20	158	12.5
5	17.5	27	20	49	17.5	71	15	93	20	115	20	137	30	159	17.5
6	12.5	28	12.2	50	20	72	20	94	25	116	20	138	20	160	15
7	7.5	29	15	51	15	73	17.5	95	20	117	20	139	20	161	17.5
8	17.5	30	22.5	52	17.5	74	20	96	22.5	118	20	140	17.5	162	15
9	10	31	10	53	12.5	75	20	97	20	119	15	141	17.5	163	12.5
10	15	32	15	54	20	76	17.5	98	30	120	17.5	142	22.5	164	22.5
11	12.5	33	15	55	15	77	12.5	99	12.5	121	25	143	7.5	165	15
12	20	34	17.5	56	15	78	20	100	17.5	122	22.5	144	30	166	25
13	15	35	9.5	57	20	79	20	101	10	123	17.5	145	20	167	17.5
14	15	36	7.5	58	15	80	22.5	102	15	124	15	146	20	168	20
15	12.5	37	15	59	20	81	15	103	17.5	125	12.5	147	22.5	169	15
16	10	38	15	60	17.8	82	15	104	15	126	12.5	148	22.5	170	15
17	15	39	15	61	17.5	83	20	105	27.5	127	22.5	149	45	171	20
18	12.5	40	15	62	17.5	84	20	106	22.5	128	22.5	150	20	172	20
19	10	41	20	63	17.5	85	20	107	22.5	129	15	151	22.5	173	12.5
20	10	42	17.5	64	15	86	20	108	15	130	20	152	17.5	174	17.5
21	17.5	43	20	65	17.5	87	20	109	20	131	22.5	153	20	175	20
22	15	44	12.5	66	20	88	17.5	110	17.5	132	17.5	154	7.5		
**Apparently-Healthy (3**^**rd**^ **Peak)**															
**Leaf#**	**Width (µm)**	**Leaf#**	**Width (µm)**	**Leaf#**	**Width (µm)**	**Leaf#**	**Width (µm)**	**Leaf#**	**Width (µm)**	**Leaf#**	**Width (µm)**	**Leaf#**	**Width (µm)**	**Leaf#**	**Width (µm)**
176	0	183	20	190	12.5	197	0	204	0	211	0	218	15	225	12.5
177	10	184	0	191	15	198	17.5	205	0	212	0	219	0	226	20
178	0	185	15	192	0	199	12.5	206	10	213	0	220	10	227	0
179	17.5	186	17.5	193	10	200	15	207	15	214	12.5	221	0	228	0
180	0	187	12.5	194	17.5	201	0	208	0	215	0	222	0		
181	12.5	188	15	195	0	202	12.5	209	0	216	0	223	15		
182	0	189	15	196	0	203	0	210	17.5	217	15	224	0		
**Infected (3**^**rd**^ **Peak Width)**															
**Leaf#**	**Width (µm)**	**Leaf#**	**Width (µm)**	**Leaf#**	**Width (µm)**	**Leaf#**	**Width (µm)**	**Leaf#**	**Width (µm)**	**Leaf#**	**Width (µm)**	**Leaf#**	**Width (µm)**	**Leaf#**	**Width (µm)**
232	0	234	0	236	0	238	0	240	0						
233	0	235	0	237	0	239	0	241	0						

## Technical Validation

**Fig. 10:**
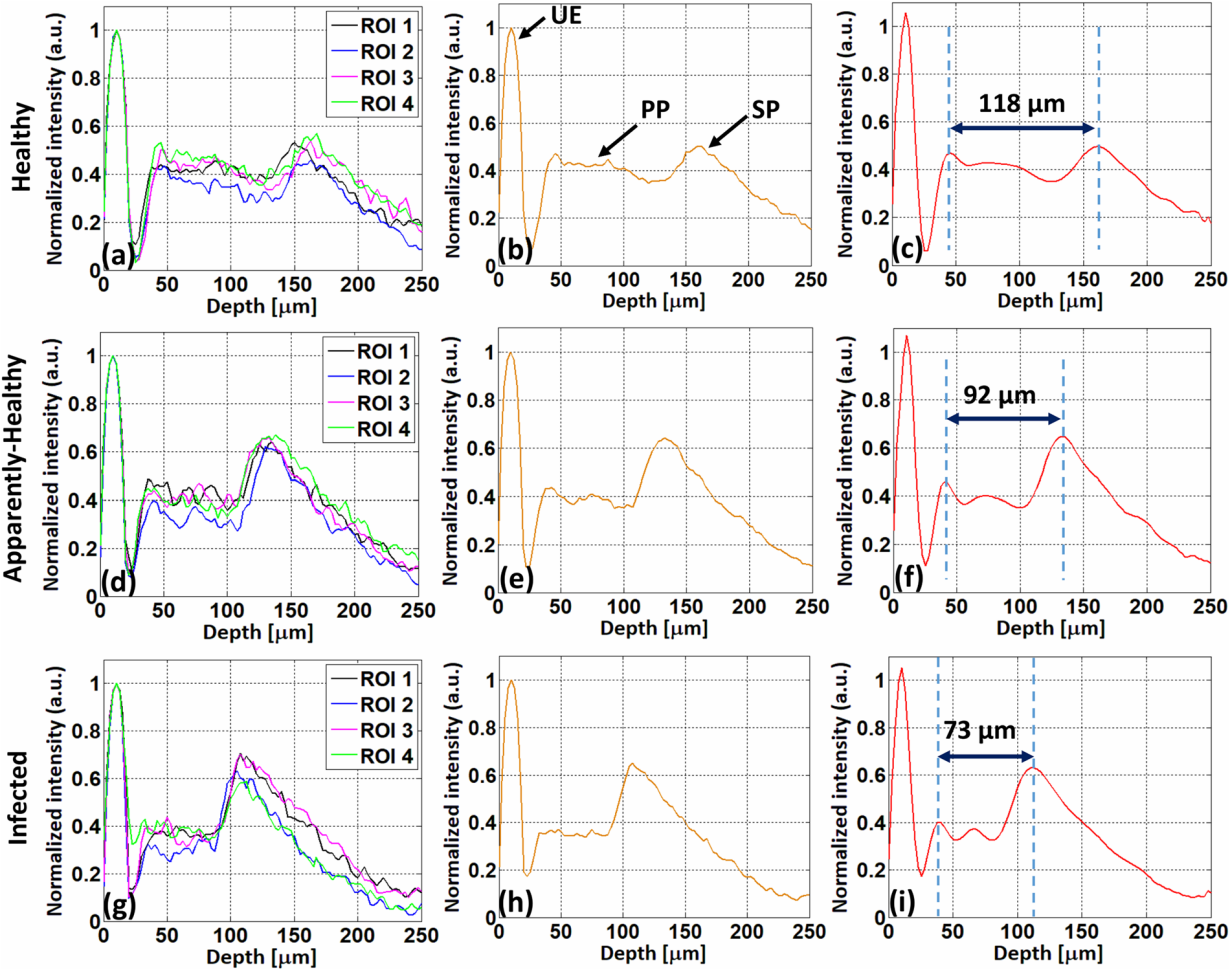
Depth profiles of healthy, apparently-healthy, and infected persimmon leaves. (**a**–**c**), (**d**–**f**), and (**g**–**i**) show depth profiles of healthy, apparently-healthy, and infected persimmon leaves, respectively. (**a**,**d**, and **g**), (**b**,**e**,and **h**), and (**c**,**f**, and **i**) show the depth profiles of four regions of interest (ROIs), average depth profiles of four ROIs, and curve-fitted depth profiles of four ROIs of a single persimmon leaf, respectively. UE: upper epidermis, PP: palisade parenchyma, SP: spongy parenchyma.

Our results can be technically validated using depth intensity profile analysis of persimmon and apple leaves. Depth intensity profiles of healthy, apparently-healthy, and infected persimmon leaves are shown in Fig. 10(a–c), Fig. 10(d–f), and Fig. 10(g–i), respectively. Black, blue, magenta, and green represent the depth intensity profiles of four ROIs of a single persimmon leaf. Figure 10(a,d,g) shows the depth intensity profiles of four ROIs of a leaf from healthy, apparently-healthy, and infected persimmon plants, respectively. The algorithm for obtaining the depth intensity profile of a single leaf is discussed in Section 2.2. Figure 10(b,e,h) shows the average depth intensity profiles of four ROIs from single healthy, apparently-healthy, and infected persimmon leaves, respectively. Finally, the curve-fitted depth intensity profiles reveal the thickness difference of the PP layer in healthy, apparently-healthy, and infected persimmon leaves [Fig. 10(c,f,d,i, respectively)]. Based on the curve-fitted depth intensity profile of four ROIs of a single leaf, values of PP layer thicknesses of healthy, apparently-healthy, and infected persimmon leaves were 118, 92, and 73 µm, respectively. PP layer thickness gradually decreased from healthy to apparently-healthy and infected persimmon leaves, where the UE and SP layers were the same for all.

**Fig. 11:**
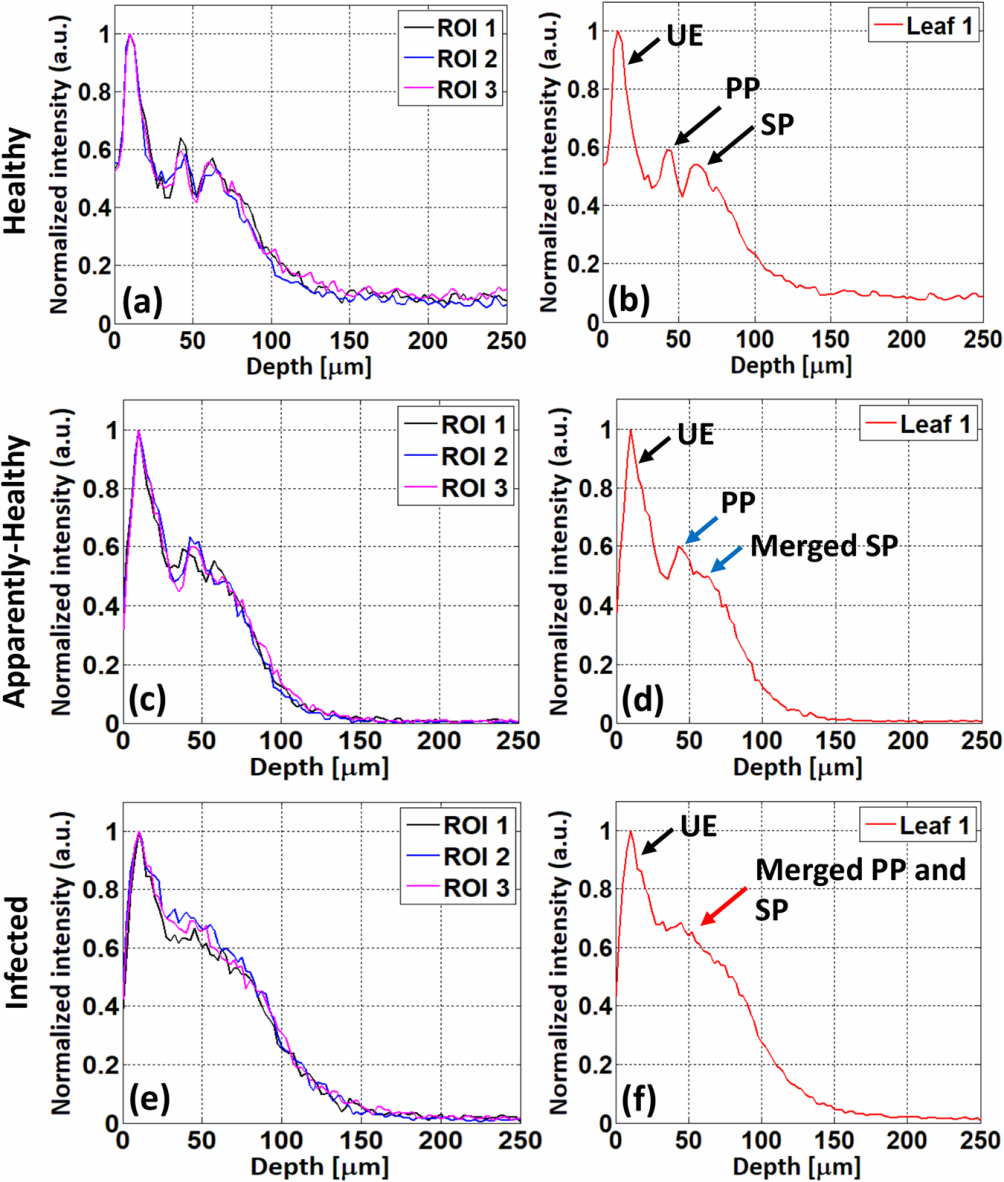
Depth profiles of healthy, apparently-healthy, and infected apple leaves. (**a**, and **b**), (**c**, and **d**), and (**e**, and **f**) show depth profiles of healthy, apparently-healthy, and infected apple leaves, respectively. (**a**,**c**, and **e**) depth intensity profiles of three regions of interest (ROIs) from single healthy, apparently-healthy, and infected apple leaves, respectively. (**b**,**d**, and **f**) averaged depth intensity profiles of three ROIs of a single leaf of healthy, apparently-healthy, and infected apple leaves, respectively. UE: upper epidermis, PP: palisade parenchyma, SP: spongy parenchyma.

Depth intensity profiles of healthy, apparently-healthy, and infected apple leaves are shown in Fig. 11(a–f), respectively. Black, blue, and magenta represent depth intensity profiles of three ROIs from a single apple leaf. Figure 11(a,c,e) show depth intensity profiles of three ROIs from healthy, apparently-healthy, and infected apple leaves, respectively. The algorithm for obtaining the depth intensity profile of a single leaf is described in Section 2.3. Figure 11(b,d,f) shows average depth intensity profiles of three ROIs from single healthy, apparently-healthy, and infected apple leaves, respectively. In the average depth intensity profile of a healthy leaf, shown in Fig. 11(b), three distinguishable intensity peaks can be visualized, which show the presence of three distinct layers in a healthy leaf, indicated by black arrows. In the average depth profile of an apparently-healthy leaf, shown in Fig. 11(d), the intensity peaks of the PP and SP layers merged (blue arrows) to appear as a single peak, which is separate from the UE layer peak. Finally, in the average depth profile of an infected apple leaf, shown in Fig. 11(f), the intensity peaks of the UE, PP, and SP layers merge (red arrow) and appear as a single peak.

## Supplementary information

Supplementary file for MIAPPE checklist

## Data Availability

Two separate MATLAB programs were used for flattening the captured OCT images and for A-scan profiling. MATLAB version 8.3 (R2014a) was installed for running the script and there was no special requirement for this analysis. First, the OCT image needs to be loaded in the image flattening program, and then the mouse pointer needs to be dragged from the right to the left of the image to get the desired flattened image. The ROI of the flattened section can be adjusted. After saving the flattened OCT images, the A-scan profiling program needs to be applied to the saved flattened images to get their A-scan profiles. The details documentation of the MATLAB script will help to reuse the code. From A-scan profiles, the width and height of UE, PP, SP layer intensity, and leaf thickness can be measured to estimate and set the threshold for pre-identification of leaf abnormalities. The leaf layer intensity and thickness measurement process has already been discussed in the ‘leaf inspection algorithm for persimmon and apple’ section in the methods part. The MATLAB program files named ‘Image_flattening.m’ and ‘A_scan_profiling.m’ have been uploaded to Figshare^63^.
