# Supplementary material for: On-field optical imaging data for the pre-identification and estimation of leaf deformities
Source: Sci Data. 2022 Nov 12;9:698. doi: 10.1038/s41597-022-01795-4 (PMC9653404; doi:10.1038/s41597-022-01795-4)
Supplement: Supplementary file 1 — Supplementary file for MIAPPE checklist [file 41597_2022_1795_MOESM1_ESM.docx]

Table: MIAPPE checklist for plant phenotyping data.

| **MIAPPE Checklist** | **Value** |
| --- | --- |
| **Investigation** | |
| Investigation title | On-field optical imaging data for the pre-identification and estimation of leaf deformities |
| Investigation description | Optical coherence tomography (OCT) based imaging was used to analyze the thickness of palisade parenchyma (PP) and spongy parenchyma (SP) for determining early-stage abnormalities of leaf specimens. The qualitative and quantitative results revealed changes and abnormalities in leaf morphology in addition to disease incubation in both apple and persimmon leaves. |
| MIAPPE version | 1.1 |
| Associated publication | <https://doi.org/10.1364/AO.56.00D108> |
| **Study** | |
| Study title | On-field optical imaging data for the pre-identification and estimation of leaf deformities |
| Study description | OCT-based imaging was used to analyze the thickness of palisade parenchyma (PP) and spongy parenchyma (SP) of persimmon and apple leaf specimens. A total of 600 OCT images from 150 persimmon leaves (4 from each leaf), and 723 OCT images from 241 apple leaves (3 from each leaf) were used to set a threshold for the pre-identification of leaf abnormalities. |
| Start date of study | 7/20/2016 (persimmon), 04/05/2017 (apple) |
| End date of study | 9/5/2016 (persimmon), 06/27/2017 (apple) |
| Contact Institution | Kyungpook National University, Daegu, South Korea |
| Geographic location (country) | South Korea |
| Geographic location (latitude) | 35.8714354 |
| Geographic location (longitude) | 128.601445 |
| Geographic location (altitude) | 1,058 m |
| Observation unit level hierarchy | field>tree>leaf->ROI (region of interest) |
| Observation unit description | On every experiment day, five trees were selected from one persimmon field for OCT imaging, and 3 leaves from each tree were imaged. From each leaf, four regions of interest positions were imaged. On every experiment day, five trees were selected from one apple field for OCT imaging, and 3 leaves from each tree were imaged. From each leaf, three regions of interest positions were imaged. |
| **Person** | |
| Person name | Ruchire Eranga Wijesinghe, Seung-Yeol Lee |
| Person email | erangawijesinghe@sjp.ac.lk, leesy1123@knu.ac.kr |
| Person ID | Ruchire Eranga Wijesinghe: orcid.org/0000-0003-2271-1885,  Seung-Yeol LEE: orcid.org/0000-0003-1676-0330 |
| Person role | author |
| Person affiliation | Department of Materials and Mechanical Technology, Faculty of Technology, University of Sri Jayewardenepura, Pitipana, Homagama, 10200, Sri Lanka. School of Applied Biosciences, Kyungpook National University, 80, Daehak-ro, Buk-gu, Daegu 41566, South Korea. |
| **Data File** | |
| Data file link | https://figshare.com/s/ed7cd56e57b1d060055c |
| Data file description | OCT images and MATLAB programs |
| Data file version | 1.0 |
| **Biological Material** | |
| Organism | Apple: NCBI TAXON: 3750 Persimmon: NCBI TAXON: 35925 |
| Genus | Malus Diospyros |
| Species | domestica kaki |
| Infraspecific name | cultivar: 'Fuji' cultivar: 'Sangjudungsi' |
| **Event** | |
| Event type | Leaf OCT imaging |
| Event description | OCT imaging of persimmon and apple leaves with backpack-type OCT imaging system |
| Event date | 2016-07-20T10:23:21+00:00; 2016-07-29; 2016-08-13; 2016-08-23; 2016-09-05 (persimmon); 2017-05-04T10:23:21+00:00; 2017-05-08; 2017-05-11; 2017-05-16; 2017-05-19; 2017-05-23; 2017-05-26; 2017-05-30; 2017-06-02; 2017-06-05; 2017-06-09; 2017-06-13; 2017-06-16; 2017-06-20; 2017-06-23; 2017-06-27 (apple) |
| **Observation** **Unit** | |
| Observation unit ID | F1T1L1R1 (F=field, T=tree, L=leaf, R=ROI) |
| Observation unit type | leaf's cross-sectional image |
| **Sample** | |
| Sample description | The leaf's adaxial side was imaged, and the 2D cross-sectional image length was 2.2 mm. |
| Collection date | 2016-07-20T10:23:21+00:00; 2016-07-29; 2016-08-13; 2016-08-23; 2016-09-05 (persimmon); 2017-05-04T10:23:21+00:00; 2017-05-08; 2017-05-11; 2017-05-16; 2017-05-19; 2017-05-23; 2017-05-26; 2017-05-30; 2017-06-02; 2017-06-05; 2017-06-09; 2017-06-13; 2017-06-16; 2017-06-20; 2017-06-23; 2017-06-27 (apple) |
| **Observed Variable** | |
| Method | Apple and Persimmon Leaf Monitoring using OCT |
| Method description | 850 nm wavelength range Wearable OCT system. 150 persimmon and 241 apple leaves were used to set a threshold for the pre-identification of leaf abnormalities. |
| Reference associated to the method | <https://doi.org/10.1364/AO.56.00D108> |
| Scale | µm |
| **Environment** | |
| Air temperature | Persimmon: 23°C Apple: 21°C |
| **Experimental Factors** | |
| Fungicide regime | Two treatment until sample collection: Mancozeb; (40g / 20L), after two weeks, Cyprodinil+Difenoconazole; (10ml / 20L) |
| Pesticide regime | Glufosinate ammonium (300mL) / water (100L) for 10a |
